# Ten Prize Hygienic Rules

**Published:** 1899-04

**Authors:** 


### Ten Prize Hygienic Rules.

The following maxims won a prize offered in 1897 by the Parisian publishers, Hatchette & Company, for the ten most effectual rules for the preservation of mental and bodily health. The author, Dr. Decornet, of Ferte-sur-Aube, won over five hundred competitors. The rules, as translated in *The Lancet*, run thus:

1. General Hygiene: Rise early, go to bed early, and in the meantime keep yourself occupied.

2. Respiratory Hygiene: Water and bread sustain life, but pure air and sunlight are indispensable for health.

3. Gastro-intestinal Hygiene: Frugality and sobriety are the best elixir for a long life.

4. Epidermal Hygiene: Cleanliness preserves from rust; the best kept machines last longest.

5. Sleep Hygiene: A sufficiency of rest repairs and strengthens; too much rest weakens and makes soft.

6. Clothes Hygiene: He is well clothed who keeps his body sufficiently warm, safe guarding it from all abrupt changes of temperature while at the same time maintaining perfect freedom of motion.

7. House Hygiene: A house that is clean and cheerful makes a happy home.

8. Moral Hygiene: The mind reposes and resumes its edge by means of relaxation and amusement, but excess opens the door to the passions and these attract the vices.

9. Intellectual Hygiene: Gaiety conduces to love of life and love of life is the half of health; on the other hand, sadness and gloom help on old age.

10. Professional Hygiene: Is it your brain that feeds you? Don't allow your arms and your legs to become ankylosed. Dig for a livelihood, but don't omit to burnish your intellect and elevate your thoughts.

